# Clitoridectomy

**Published:** 1867-04

**Authors:** 


Clitoridectomy.—Mr. Baker Brown and Mr. Philip Harper, in deference to the opinion of the medical press on the subject of clitoridectomy, have determined not to perform the operation in the London Surgical Home, pending professional inquiry into its validity as a scientific and justifiable operation. What now will be the chance of recovery for the poor epileptic female with a clitoris?—*Medical Record.*

